# Metritis

**Published:** 1895-04

**Authors:** Robert T. Morris

**Affiliations:** Houston, Texas


For Texas Medical Journal.

### METRITIS.

BY ROBT. T. MORRIS, M. D., HOUSTON, TEXAS.

[Read before the Houston District Medical Society.*]

IN SELECTING metritis for discussion, I feel some trepidation; first, because it is a subject about which so much can be written, and my time is limited, and, second, because of a realization of my inability to thoroughly grasp the subject and place it before you in a rational light.

The uterus is the portal for, or the cause of the great majority of pelvic diseases. Through it the gonococcus enters, producing acute inflammation, or remains latent or quiescent until traumatism or excessive venery causes it to break forth with renewed energy, leaving its impress in the form of endometritis, pustules, and pelvic peritonitis, or uncleanly midwifery and careless operators introduce the septic germs which produce the train of symptoms so well known; consequently the important position the uterus occupies in relation to pelvic troubles makes it necessary to as clearly as possible comprehend its diseases. I venture the statement that any inflammatory condition of the ovary, any salpingitis, pelvic-peritonitis or perimetritis, in the great majority of cases, can be traced, directly or indirectly, to some previous or present uterine disorder. Pozzi has the following to say: "I do not think there are any cases of ovaritis, properly so-called, that are not preceded by endometritis. It is true that one or the other of these conditions may have existed without leaving any permanent trace, but a study of previous symptoms will prove that they did exist." Scott, of Washington, says "Clinically,

---

*The TEXAS MEDICAL JOURNAL is the offiical organ of the Society.

an almost constant feature of pelvic-peritonitis is salpingitis, and the septic salpingitis follows upon a septic endometritis.''

Before entering into the question, it may not be amiss to briefly review the histological anatomy of the uterus and its adnexa. The tubes and uterus are formed from the ducts of Müller, which connect with the anterior part of the segmental body, or pronephros. The ducts passing downward and inward coalesce and form the uterus, that portion above remaining as the fallopian tubes. Thus it will be seen that the uterus and tubes are embryologically the same, and that there is a direct continuation of mucous and muscular tissues. This fact should be borne in mind, for it will explain the etiology of many tubal troubles. You all know how intimate the anastomosis is between the uterine and ovarian vessels, and that there is a chain of lymphatics at the angle of the uterus, passing behind and below the tubes and below the ovary and tubes, and also that the net work of lymphatics which cover the surface of the ovary communicate with the lymphatics of the peritoneum. The mucous membrane of the body of the uterus is covered with columnar ciliated epithelium, and contains the uricular glands, while that of the cervix is covered with squamous epithelium, and contains mucoid glands, which, under pathological conditions, become the ovules of Naboth.

The term metritis is used in its broadest sense,—uterine inflammation. While we can not arbitrarily draw the line, and say, here endometritis begins, and there ends, because a morbid condition of the mucous membrane necessarily affects the muscular layer, and vice versa, and while the line of demarcation between cervical and corporeal inflammation is not distinct, the preponderance of one of these conditions justifies their separation.

We will first consider corporeal inflammation. I will not mention the multitudinous classifications, but will adopt the simplest —acute inflammation and chronic inflammation.

### ACUTE INFLAMMATORY METRITIS.

Without question, the cause of this condition is septic infection; although there are a few cases of auto-infection, the greater part are hetero-infection. As Winter, Hausmann, and others, have shown that pathogenic germs are always present in the cervix and vagina, and although they may be in a latent or attenuated condition, we can easily understand how auto-infection could supervene, or how a favorable medium for their propagation, such as a retained placenta or gravid uterus would cause them to regain their usual virulence. Unclean hands and septic instruments are the usual methods of producing hetero-infection. Many a case of uterine trouble and diseased aduexa can be laid at the door of the gynecologist who, either from ignorance or criminal indifference, observes not the sacred law of asepsis and antisepsis. Another prolific cause of this condition is the gonococcus. While perhaps Noeggerath exaggerated somewhat the sequelæ and persistence of gonorrhœa in the female, when he stated that out of one hundred women whose husbands had contracted gonorrhœa previous to marriage, ninety would suffer with some form of pelvic trouble, and the remaining ten would be liable at any time to acute gonorrhœal inflammation, nevertheless, the gonococcus as an etiological factor in acute pelvic troubles occupies a very prominent place. An ill-fitting pessary, and especially a stem pessary, may give rise to acute symptoms of metritis, which generally disappear upon removal of the cause. Again, any mechanical condition of the uterus which produces venous stasis, as sub-involution, displacement, stenosis of cervix, incomplete development, or laceration, may at the menstrual period, or from exposure, predispose to this condition; but they more frequently produce chronic metritis.

The symptoms are those resulting from any acute infection with septic absorption, such as rigors, fever, local pain, throbbing and redness; in addition, there may be tubal disease, with inflammation of the adjacent tissues, and a puro-sanious discharge from the womb.

The pathogeny is as follows: "The uterine tissue is increased in volume, and softened, with a red color mottled with yellow. The inflammation of the mucous membrane is of an interstitial type. The glands are unchanged, but there is a peculiar alteration in the inter-glandular tissue, the cells being far more numerous than usual, and so tightly packed against each other that there is none of the homogenous intercellular substance left."

### TREATMENT.

The same general rules will apply here as in any morbid condition—remove the cause and treat the result. If the source of the trouble is a retained placenta or engrafted villi, remove and curet; if gonorrhœa is present, vaginal and intra-uterine injection of nitrate of silver or bichloride of mercury will generally suffice, if not, the probabilities are strong that there is a mixed lesion, and curettage is necessary. In cases of acute metritis with tubal involvement, except fluid tumors, curettment is positively indicated; that is, in cases of purulent salpingitis and perimetritis. In the American Text-Book of Gynecology you will find the following: "In the light of its causation and pathology, curettage is positively indicated in every case of tubal and peritoneal trouble, when there is a suspicion that the infection originated in the endometrium. Then one of three methods must be adopted; either poultices and hot douches, curettment and treatment of the uterus as any septic cavity, or a primary cœlotomy. The first is the method of the midwife, and merely allows the infection to work its will in the pelvis; the second is surgical in every sense of the word, while to adopt the third in every case stamps a man as blind to reason and to the work of other men, and willing to open a fellow-being's abdomen rashly and unnecessarily."

That seems somewhat a radical and dangerous procedure, as there are many cases where a positive diagnosis can not be made, and currettage under those circumstances might rupture a pus tube and produce a fatal termination; but if the diagnosis is certain,—and a careful examination, under the influence of chloroform, should always be made,—the most logical and rational method to pursue is to remove the source of contamination by currettage, and by such means many cases of non-cystic salpingitis and pelvic peritonitis can be cured before they become a permanent pathological entity. There are cases where currettage is not so necessary: such as those comparatively mild in character, which occur at the menstrual period, and complicated with uterine malposition, and also those due to exposure or excessive venery. Rest, salines, hot douches, and cervical punctures, will answer every purpose.

### CHRONIC METRITIS.

This condition, pathologically, may be divided in the following classes: Interstitial metritis, hypertrophic and hyperplastic, glandular metritis and sclerotic metritis.

### INTERSTITIAL METRITIS.

Pozzi, in speaking on interstitial metritis says: "the interglandular tissue which you have seen gorged with cells in the acute form so that it resembled granulation tissue is transformed into true cicatricial tissue in which the number of cellular elements steadily increase. The glands undergo the opposite alteration, being strangulated in places and transformed into cysts are so compressed in their whole extent, that they artophy, and thus we may have a few glands scattered through the connected tissue altered into cysts in places or totally destroyed.'' Or in other words, we have a true connective tissue sclerosis. Sometimes there are no glands present, and connective tissue is substituted for the normal mucous tissue. The most common malignant disease of the uteri of young women is sarcoma of the endometrium, which is always preceded by an interstitial inflammation. Abortive endometritis and membraneous dysmenorrhœa are types of interstitial inflammation. Hemorrhage is quite a constant feature of this condition.

### GLANDULAR METRITIS.

Wyder recognized the two forms of the glandular metritis, the hypertrophic and the hyperplastic. In the former there is an epithelial proliferation with multiplication of glands; in the latter there is an increase in the number of glands. Between the interstitial and glandular type we find the polypoid type, which partakes of both, with a large increase of mucous tissue. This is the fungous or vegetative type.

There are also uteri characterized by hypertrophic or the later stage sclerotic conditions. There is an enlargement of the uterine wall due to increase of connective tissue between the muscles and around the vessels. It may remain thus, forming the hypertropic form, or the connective tissue may contract and become sclerotic.

### TREATMENT.

There are some cases of metritis due to venous stasis dependent upon displacements, stenosis, etc., others due to constitutional causes which can be cured by rest, pessaries, douches, tampons and tonics. But when there are far advanced anatomical changes, nothing but the curette will cure, and sometimes that will fail; especially in those cases with large lacerated cervices, when trachelorrhaphy or amputation of the cervix must be resorted to.

### CURRETTMENT.

Polk says, "Of all conservative operations upon the uterus the simplest and the farmost reaching in its results is currettage with drainage and depletion which is obtained by the plentiful packing of its cavity with sterilized gauze.'' Quite a number of operators differ as to the technique of the operation. Instead of mentioning the points of difference, I will hurriedly relate the manner in which I would perform it.

The operation should be performed the first few days after menstruation, and it is advisable in our southern climate to put the patient on small doses of quinine a few days previous. A large saline should be given from sixteen to twenty-four hours previous, and an enema the morning of the operation, and if you are ultra aseptic, the rectum may be washed with a boracic or salicylic acid solution. The vagina should be douched with bichloride of mercury, one to three thousand, thoroughly taking special precaution to cleanse the interior and posterior cul-de-sac and the vaginal portion of cervix so as to remove the germs that always exist in those localities. The douches should be given three hours previous to, and again immediately before operating. Pre-supposing that the rules of asepsis and antisepsis are observed, the cervix is well dilated; although some say "it is illusory as regards the escape of secretions, and that it lasts but a few hours." After dilatation a dull curette in a gravid uterus,—Thomas' for instance, but a sharper one in other cases is used, previously ascertaining the depth and direction of the cavity with the uterine sound. The posterior and anterior face, the fundus, the angles, and the sides, are scraped in the order named. It is well to steady the cervix with vulcella forceps. After curretting, the cavity is flushed with a double current catheter, using mildly carbolized or sterilized water; then cauterized with saturated solution of carbolic acid, reflushed and packed with iodoformed gauze. The gauze packing is a very essential feature, and as Polk says, "there are few cases of chronic uterine inflammation in which depletion is not demanded; wherever any degree of involvement of the wall is present it is a necessity, and nothing gives this better than gauze. Pack it in place firmly and abundantly, and apart from the drainage, which it secures, it will force the uterus into contractile efforts, and produce the disintegration and absorption of the adventitious tissue.

The completion of the operation does not terminate the case, as some seem to think, for the predisposing cause quite frequently remains, which should be removed. And again, there are hard, woody, sclerotic uteri, which do not yield to any treatment but hysterectomy, which should be the last resort. I will only mention a few words in regard to morbid changes in the cervix. The cervical changes generally depend upon laceration or corporeal diseases; hence Emmet's operation or currettage will cure the case.

In conclusion I will say, if we correct displacements, repair lacerations, remove fœtal debris, and insist upon and practice obstetrical and gynecological asepsis and antisepsis, metritis and perimetritis, salpingitis and ovaritis, and pelvic peritonitis will become pathological rarities, and the sneering remark "once in the hands of the gynecologist always in the hands of the gynecologist" will react to the discredit of the promulgator; or to be more concise, take care of the uterus, and the adnexa will take care of themselves.

